# A qualitative study on acceptability of the mistreatment of women during childbirth in Myanmar

**DOI:** 10.1186/s12978-020-0907-2

**Published:** 2020-04-20

**Authors:** Thae Maung Maung, Kyaw Lwin Show, Nwe Oo Mon, Özge Tunçalp, Nyein Su Aye, Yin Yin Soe, Meghan A. Bohren

**Affiliations:** 1 Department of Medical Research, Ministry of Health and Sports, No.5, Ziwaka Road, Dagon Township, Yangon, 11191 Myanmar; 2 UNDP/UNFPA/UNICEF/WHO/World Bank Special Programme of Research, Development and Research Training in Human Reproduction (HRP), Department of Sexual and Reproductive Health and Research, World Health Organization, Avenue Appia 20, 1202 Geneva, Switzerland; 3 Department of Obstetrics and Gynaecology, University of Medicine (1), Yangon, Myanmar; 4 Gender and Women’s Health Unit, Centre for Health Equity, Melbourne School of Population and Global Health, University of Melbourne, Carlton, Victoria 3053 Australia

**Keywords:** Maternal health, Childbirth, Quality of care, Mistreatment, Respectful maternity care, Gender analysis, Qualitative research, Myanmar

## Abstract

**Background:**

Improving the quality of maternal health care is critical to reduce mortality and improve women’s experiences. Mistreatment during childbirth in health facilities can be an important barrier for women when considering facility-based childbirth. Therefore, this study attempted to explore the acceptability of mistreatment during childbirth in Myanmar according to women and healthcare providers, and to understand how gender power relations influence mistreatment during childbirth.

**Methods:**

A qualitative study was conducted in two townships in Bago Region in September 2015, among women of reproductive age (18–49 years), healthcare providers and facility administrators. Semi-structured discussion guides were used to explore community norms, and experiences and perceptions regarding mistreatment. Coding was conducted using athematic analysis approach and Atlas.ti. Results were interpreted using a gender analysis approach to explore how power dynamics, hierarchies, and gender inequalities influence how women are treated during childbirth.

**Results:**

Women and providers were mostly unaccepting of different types of mistreatment. However, some provided justification for slapping and shouting at women as encouragement during labour. Different access to resources, such as financial resources, information about pregnancy and childbirth, and support from family members during labor might impact how women are treated. Furthermore, social norms around pregnancy and childbirth and relationships between healthcare providers and women shape women’s experiences. Both informal and formal rules govern different aspects of a woman’s childbirth care, such as when she is allowed to see her family, whether she is considered “obedient”, and what type of behaviors she is expected to have when interacting with providers.

**Conclusions:**

This is the first use of gender analysis to explore how gender dynamics and power relations contribute to women’s experiences of mistreatment during childbirth. Both providers and women expected women to understand and “obey” the rules of the health facility and instructions from providers in order to have better experiences. In this way, deviation from following the rules and instructions puts the providers in a place where perpetrating acts of mistreatment were justifiable under certain conditions. Understanding how gender norms and power structures how women are treated during childbirth is critical to improve women’s experiences.

## Plain English summary

In low to middle income countries, 300,000 women per year die from complications during pregnancy and childbirth. In Myanmar, a country in Southeast Asia, there have been big improvements in maternal mortality. However, there are still maternal health challenges in Myanmar; for example, about one-third of women give birth in a health facility. About 60% of births take place with a skilled attendant (midwife) also occurred at home. Most maternal mortality and poor maternal health can be avoided with access to good quality maternal health services. Global research evidence has shown that women may avoid maternal health services if they perceive them to be of low quality or expect to be treated poorly by healthcare providers. In this study, we used qualitative research methods (in-depth interviews and focus group discussions) with women who recently gave birth, and doctors, nurses and healthcare administrators in selected health facilities in Myanmar. We were interested to explore whether women and healthcare providers viewed different types of mistreatment to be acceptable behaviors or not. Specifically, we were interested in exploring providers pinching or slapping, shouting at, physically restraining, or refusing to help a woman while she is in labor. Using qualitative methods, we are able to understand people’s personal experiences and social norms around how women are treated during childbirth. We also used a gender analysis approach to interpret the results. The gender analysis approach helped us to explore how power dynamics, hierarchies, and gender inequalities may influence how women are treated during childbirth. We found that women and providers were mostly unaccepting of the different types of mistreatment. However, some provided justification for slapping and shouting at women, if these behaviors were used to “help the woman”, for example by encouraging her to push or motivating her. We also found that different types of access to resources, such as financial resources, information about pregnancy and childbirth, and support from family members during labor may impact how women are treated. Furthermore, social norms around pregnancy and childbirth and relationships between healthcare providers and women may shape women’s experiences. Lastly, both informal and formal rules may govern different aspects of a woman’s childbirth care, such as when she is allowed to see her family, whether she is considered to be obedient, and what type of behaviors she is expected to have when interacting with the providers. These results will inform discussions in Myanmar about how maternal health services may be structured and organized in a way that helps both women and providers to have good experiences.

## Introduction

Low and middle-income countries (LMICs) account for 99% of worldwide maternal deaths - approximately 302,000 in 2015 - most of which can be prevented with access to timely, safe and effective care [1, 2]. In Myanmar, the maternal mortality ratio (MMR) has declined from 340 to 250 maternal deaths per 100,000 live births between 2000 and 2017, however, it is still high in comparison to other Asian countries [3]. Key components to reduce maternal mortality and improve maternal health are to ensure that every woman gives birth in the presence of a skilled birth attendant and encourage facility-based childbirth. In low resource settings, perceptions of poor quality of care are a major contributing factor affecting low utilization of maternal health care services and high rates of mortality [4]. Improving maternal health therefore not only needs to address economic, cultural and geographical challenges, but also promote quality of care [5]. In order to increase the proportion of births with skill birth attendants and facility-based childbirth, both coverage and quality of care provided to women need to be improved [6].

Although there have been global increases in maternal health, maternal mortality remains high, along with the presence of poor quality care and mistreatment during childbirth [4, 7]. After the 1994 International Conference on Population and Development in Cairo, Egypt, human rights aspects were considered to provide quality of care, which is comprised of both the context of clinical care, standardized skills and interpersonal relationships between healthcare workers and women [8]. More recently, the World Health Organization (WHO) vision for quality of care for women and newborns emphasizes the importance of both the ‘provision of care’ and ‘experience of care’ [9]. Recent studies have shown that women experience mistreatment during childbirth in health facilities, including disrespectful, neglectful and abusive care [10–13]. This mistreatment can be an important barrier for women when considering facility-based childbirth [4]. Furthermore, the WHO standards for improving quality of maternal and newborn care in health facilities highlight the importance of effective communication that is responsive to specific needs and preferences of women and their families, providing care with respect and dignity, staff’s motivation and competency, and appropriate physical environment as critical components of quality care [14].

### WHO study: “How women are treated during childbirth”

A mixed-methods systematic review by Bohren and colleagues developed a typology of the mistreatment of women during childbirth which included: physical, sexual and verbal abuse, stigma and discrimination, failure to meet professional standards of care, poor rapport between women and providers and health systems conditions and constraints [15]. In response to growing recognition that women are mistreated during childbirth around the world, WHO launched a multi-country study on “How women are treated during childbirth.” The study used a mixed-methods design to understand and measure the mistreatment of women during childbirth in Myanmar, Nigeria, Ghana and Guinea. In the first phase, formative qualitative research was conducted to understand women’s and providers’ experiences and acceptability of mistreatment during childbirth [16–21]. The formative findings and systematic review [15] were used to inform the development of two measurement tools in the second phase (direct observations of labor and childbirth) and a community-based survey with women during the postpartum period in the same settings [22, 23].

This paper explores the acceptability of mistreatment during childbirth in Myanmar according to women and healthcare providers, and uses a gender analysis framework to understand how gender power relations influence mistreatment during childbirth. This analysis thus contributes to understanding why mistreatment during childbirth may occur in Myanmar and how gendered power dynamics may influence how women are treated.

## Methods

### Study context and sites

Myanmar has made progress on improving maternal health, with a 60.7% improvement in the MMR between 1990 and 2015 [1]. However, MMR is still high in comparison to other regional countries [24]. According to 2015–16 Myanmar Demographic and Health Survey (MDHS) data, facility-based childbirth was 37%, but 60% of births were assisted by skilled attendants (nurses, midwives and doctors) [25]. In Myanmar, the skilled birth attendance rates are high compared to facility-based childbirth, because some cadres of skilled birth attendants (midwives and lady health visitors) also attend home births, particularly in rural areas. The total fertility rate (TFR) in Myanmar is 2.3 children per women, 1.9 in urban and 2.4 in rural areas [25]. Contraceptive prevalence rate (modern methods) is 51% and unmet need for family planning among currently married women is 16% [25]. Approximately 60% of pregnant women had antenatal care coverage (four or more antenatal visits) and 71% of women had the recommended postnatal care [25]. In Myanmar, maternal health services are delivered by hospitals and maternal and child health clinics (MCH) in urban area and rural health centers (RHCs) and sub-health centers in rural areas. Maternal health services in rural areas are delivered mainly by midwives who are specially trained for maternal and child health.

This study was conducted in two townships in the Bago Region, Myanmar, where the facility-based childbirth rate was 35–40% in 2015 [25]. In Bago Region, the median age at first marriage is 21.8 years and the total fertility rate is 1.9 [25]. Study sites were purposively chosen depending on the number of births per month, number of staff in study health facilities, and an existing relationship between the selected health facilities and research organization. Table 1 presents the characteristics of the study facilities.

**Table 1:** Facility characteristics

	Facility 1	Facility 2
**Number of beds**	200	200
**Total admissions**	18,152	17,088
**Total out-patients**	40,332	47,357
**Total births**	1653	1486
**Abortions**	243	258

Source: Department of Public Health in collaboration with Department of Medical Services; Ministry of Health; The Republic of the Union of Myanmar. Annual Hospital Statistics Report 2013. Nay Pyi Taw, Republic of Union of Myanmar; 2013

### Study participants, recruitment and sampling

Three groups of participants were identified: women (identified from communities in the selected facility catchment areas), healthcare providers and facility administrators (identified from the selected facilities). First, to explore community norms regarding mistreatment during childbirth, focus group discussions (FGDs) were conducted with women of reproductive age (18–49 years) who had given birth in any facility in the last 5 years. Second, to explore individual experiences and perceptions regarding mistreatment during childbirth, in-depth interviews (IDIs) were conducted with women of reproductive age (18–49 years) who had given birth in any facility in the past 12 months. To explore experiences and mistreatment during childbirth, IDIs were conducted with healthcare providers (nurses/midwives and doctors/specialists) in each of the identified facilities. To explore the facility and health system related factors contributing to mistreatment, IDIs were conducted with the facility administrators at each facility (e.g. head of obstetrics, matron-in-charge).

The research teams facilitated contact with women in the communities in the selected facility catchment areas and the healthcare providers in the selected facilities. Women who reside in the catchment areas of the selected facilities were approached face-to-face and invited to participate in the study with the help of the midwives and their birth registers. Purposive quota sampling was utilized to achieve a stratified sample without random selection. This method uses specified parameters (i.e., setting, designations, religion, etc.) to stratify the sample. Women were sampled from urban and rural settings for FGDs and IDIs. Healthcare providers were recruited based on their cadre, such as nurses or doctors/specialists. Two facility administrators per facility were sampled.

### Study instruments

All instruments were semi-structured discussion guides, fostering comparability across IDIs/FGDs and allowing participants to guide the discussion based on their experiences. Instruments were pilot tested during a training workshop for research assistants. Women, nurses and doctors were presented with four scenarios that could be classified as mistreatment during childbirth including slapping, verbal abuse, neglect and physical restraint during labor. Research participants were then asked if the scenario was acceptable, when (if ever) it would be acceptable, and how they would feel if it happened to them (or their sister or wife in case of a male provider) [19].

### Data collection and management

Each individual was provided with information about the study, invited to participate and asked to provide consent. All FGDs and IDIs with women took place in a private setting with no one else present such as the meeting room of the school. Given the sensitivity of the topics discussed, all FGD and IDI moderators were female for the FGDs and IDIs with women. Research assistants who conducted interviews with women were female, non-clinical technicians from Department of Medical Research (DMR), Myanmar who took part in a variety of research projects conducted on different health topics. IDIs with healthcare providers and hospital administrators were conducted in a hospital meeting room where privacy was ensured. Interviews with healthcare providers and hospital administrators were conducted by trained researchers from DMR, Myanmar who were medical doctors. All the data collectors received two-day intensive training for this study, and had previous experience conducting qualitative interviews and FGDs and IDIs.

All FGDs and IDIs lasted between 60 to 90 min and were audio recorded. Participants received 7000 kyats (approximately $7 USD) and a refreshment to compensate their time. There was no additional contact with the participants after data collection was completed. Data were collected in September 2015, until thematic saturation was reached. Audio files were transcribed verbatim into Myanmar language, supplemented with field notes, then translated into English. The translated transcripts were reviewed for consistency by DMR Research Officers. Transcripts were shared and reviewed on an on-going basis to ensure data quality. De-identified transcripts were stored on password-protected computers of principal and co-investigators.

### Data analysis

A qualitative data analysis workshop was conducted for the research team facilitated by TMM and MAB. First, we used a thematic analysis approach as described by Braun and Clark for analysis [26]. The development of the code book used both inductive and deductive approaches, based on the typology of mistreatment during childbirth developed by Bohren and colleagues [15] and the interview guides (deductive), and supplemented by the themes emerging naturally from the data and from the discussion (inductive). Coding was conducted by the research team (TMM, NOM, NSA, KLS) using Atlas.ti software [Scientific Software Development GmbH. Version 6. Berlin, Germany], and outputs were generated for specific codes. Output data were categorized on the basis of their thematic similarity for further interpretation.

After the descriptive thematic analysis, we used a gender analysis framework approach [27, 28] to explore how gender dynamics and power relations contribute to women’s experiences of mistreatment during childbirth. Gender analyses is a tool to explore how social inequalities and power dynamics shape interactions with health care services and health systems [27]. Using a gender analysis framework (Table 2) allows for exploration of gender as a driver of inequality and power dynamics, including how power is constituted and negotiated through four key domains: *who has what* (access to resources), *who does what* (division of labor and everyday practices), *how values are defined* (social norms, perceptions and beliefs) and *who decides* (rules and decision-making) [27]. These domains are presented as distinct categories but are interacting with and reinforcing each other as people negotiate with others and their environments [29]. Gendered power dynamics may be challenged or remain static as people negotiate their interactions within a health system and broader society [27]. This analytic component was informed by the framework analysis approach which is a pragmatic approach using a series of steps (indexing, charting, mapping and interpretation) to organize and interpret data [28]. Transcripts, the thematic codebook, and thematic coding outputs were reviewed and organized using the gender analysis framework structure. To our knowledge, the intersection between mistreatment during childbirth, gender inequality, and clinical hierarchies has not yet been explored using a gender analysis approach. During this analysis, key new analytic findings related to gender and power emerged.

**Table 2:** Gender analysis framework: understanding gender as a power relation and driver of inequality [27]

**What constitutes gendered power relations?**	Who has what?	Access to resources (education, information, skills, income, employment, services, benefits, time, space, social capital)
	Who does what?	Division of labor within and beyond the household and every day practices
	How are values defined?	Social norms, ideologies, beliefs and perceptions
	Who decides?	Rules and decision-making (formal and informal)
**How is power negotiated and changed?**	Individuals/people	Critical consciousness, acknowledgement (or lack of), agency/apathy, interests, historical and lived experiences, resistance, violence
	Structural/environmental	Legal and policy status, institutionalism within planning and programs, funding, accountability mechanisms

Throughout the analysis process, the members of the research team considered questions related to reflexivity, including the influence of their preexisting assumptions and experiences related to childbirth upon their interpretation of the study results, and that of the results upon their perspectives.

## Results

The study was conducted from 1st to 25th September 2015 in Bago and Taungoo Townships in which 32 IDIs were conducted with women who gave birth in facility in the past 12 months, eight FGDs with women who gave birth in the last 5 years, and IDIs were conducted with 25 healthcare providers and 4 administrators. Table 3 shows the socio-demographic characteristics of women and Table 4 reports the socio-demographic characteristics of healthcare providers.

**Table 3:** Socio demographic characteristics of the participants: women of reproductive age

	IDIs (*n* = 32)	FGDs (nFGD = 8) (*n* = 72)
**Age (years)**		
20–24	5	11
25–29	8	21
30–34	6	25
35–39	9	11
40+	4	4
**Marital status**		
Single	0	0
Married	31	72
Divorced/Widowed	1	0
**Location**		
Urban	16	4
Rural	16	4
**Religion**		
Buddhist	**30**	72
Other	2	0
**Ethnicity**		
Myanmar	**28**	72
Other	4	0
**Education**		
None	0	3
Primary	11	9
Secondary	11	23
Tertiary	6	22
University/Graduate/Diploma	4	15
**Employment**		
Business/private sector/Seller	8	12
Civil servant	1	4
Daily wagers	6	3
Housewife	15	49
Others	0	4
**Number of living children**		
0–1	15	23
2–3	15	34
≥ 4	4	15

**Table 4:** Socio demographic characteristics of the participants: healthcare providers and administrators

	Nurse *n* = 16	Doctors *n* = 9	Administrators *n* = 4
**Age (years)**			
20–29	9	4	0
30–39	4	2	0
40–49	1	0	4
50+	2	3	0
**Marital status**			
Single	14	6	2
Married	2	3	2
**Gender**			
Female	16	15	3
Male	0	1	1
**Years of experience**			
0–4	11	6	0
5–9	3	0	0
10–15	0	1	4
15+	2	2	0
**Hospital**			
Facility 1	8	4	2
Facility 2	8	5	2

Most women were married and under 40 years old (92%), and well balanced between urban and rural areas. Most were housewives (63%), Buddhist (98%) and of Myanmar ethnicity (96%). Their education status varied, 52% with primary or secondary level education while 45% with tertiary level education and above (university graduate/diploma). Two-thirds of the participants had two to five children alive. Among the healthcare providers, almost all were females (94%) with four or fewer years of experience (68%) and 5 to 10 years of experience for administrators.

The following sections report the results of the four scenarios of mistreatment during childbirth, followed by the gender analysis.

### Acceptability of a provider pinching or slapping a woman

Although most women in our study had not experienced pinching or slapping during labor, women from urban areas were more accepting of this behavior than those from rural areas. This was particularly true if pinching or slapping was used to encourage women or it was done “for their sake”.

*“I could accept slapping to push. If I could not deliver, I had to push. I had to put effort. The nurses also said the prayer.”* [FGD, woman, urban]

Those who felt that pinching or slapping was unacceptable stated it made them feel sad, worried and scared. Women’s opinions were divided almost equally between those believing pinching and slapping was acceptable (*n* = 17) and unacceptable (*n* = 18).

*“If they slapped or pinched the woman in labor like that, while she was suffering from labor pain, she had to suffer from the pain in double. I would be sad then. I could not accept it.”* [IDI woman, 42 years old, urban]

From the providers’ perspective, most did not accept to pinch or slap women during labor as they viewed this type of behavior as punishment rather than encouragement or support.

*“Pinch or slap doesn’t mean an encouragement. It seems to be scolding.”*

[IDI with a nurse]

However, a few providers reported that they slapped the women during labor as an encouragement for birth in some conditions as required, for example to give them strength to persevere through labor.

*“Nothing more would happen even if we pinched or slapped. We could not tell some people ‘how to stay’. There are some people who don’t have the conscious. We can’t slap softly then. We have to slap. As she would be conscious if we slap, we have to slap a little.”* [IDI with a nurse]

Slapping or pinching in these scenarios was thus perceived to be a method used to help the woman to have a positive outcome.

### Acceptability of a provider shouting at a woman

In contrast to slapping, most of women believed that yelling or shouting by health workers during labor could not be acceptable and they would feel unhappy, sad and angry if they encountered it.

*“Respondent (R): I would not accept it. I held her as an encouragement for pushing the baby out, as I suffered from pain. She was a doctor. When she shouted me, I was sad. I was frightened.*

*Interviewer (I): How did she shout at you?*

*R: “Don’t hold me. I don’t like. Don’t hold me. Your hands were dirty, not clean.”*

*I: How did you feel then?*

*R: I held her, as I relied on her. I was sad.”* [IDI woman, 23 years old, rural]

Some answered that shouting would be acceptable because they had to rely on health workers for everything during labor or if they felt shouting it was done for their sake.

*“It would be acceptable. They were the doctors. If we were hospitalized, we had to rely on them for everything. We didn’t understand anything. It would be fine only when they did.”* [FGD woman, rural].

Women therefore reported that they depended on providers completely during labor and would be accepting of shouting if this was part of the process of giving birth in the facility. However, providers generally did not consider yelling or shouting at the women during labor to be acceptable. They reported that they may shout at the women to “push”, as an encouragement for birth, but none intended to scold the women.

*“For health workers from our hospital, there are sometimes that they do not get well with patients. If a woman in labor is asked to do this, she doesn’t obey as she is suffering from labor pain. The main thing is the way they push their legs. They do not grasp well. They do not pull well and they do not push the baby well. So, workers have to shout, “Push, push”. Nothing more. The workers have to say, “The baby is almost out, why don’t you push out”. For some, they don’t know how to push. This is not a shouting, just saying them to be good.”*

[IDI with a doctor].

The providers explained that some may be using a raised voice to encourage the woman to push rather than scolding; however, it is noted that the providers may also be raising their voices when women are perceived to be “disobedient” or not understanding guidance from the providers.

### Acceptability of a provider refusing to help a woman

Most women did not personally experience refusal to help during labor by providers, and most believed that this type of behavior was unacceptable. Women reiterated that they relied on the guidance and knowledge of providers and they would feel unhappy, sad and angry if the providers refused to help them.

*“If I asked for their help as I needed them, if they refused, I would think they had no sympathy. I would be very angry with them*.” [IDI with woman, 33 years old, rural]

Only a few responded that they would be accepting because they thought that providers may be busy with an emergency.

*“I would accept it. They may be busy with their business. They may be in an emergency. I would accept it. I would accept it during the delivery.”* [IDI woman, 25 years old, urban]

From the providers’ perspective, all the providers reported that it was unacceptable to refuse to help women during labor. However, it may depend on the type of help requested. For example, if women asked for something perceived to be impossible, they would be refused.

*“As possible as we can, we do not refuse. We explain them till they understand. We do not accept blindly everything what patient want to do. We review what they ask for. If it is necessary, we do it for her. Neither we refused all what they ask for nor accept all. If the indication and what the patients ask for are the same, we do it. Even if they refused as they don’t want to do it, we can urge them to do it.”* [IDI with a doctor]

*“If it was the thing that we could fulfill, we had to do it. If we could not fulfill, we had to refuse it. For example, if she asks us to get her attendant in, it is impossible. If she wanted to drink water, I would do it by myself.”* [IDI with a nurse]

The providers felt that they would respond to women’s requests within reason, and would only refuse a request for help if it was perceived to not be possible.

### Acceptability of physically restraining a woman

Most women were accepting of physically restraining woman during labor as they thought it was for their benefit. For example, physical restraint was viewed as a way to speed up labor and even provide some protection or encouragement.

*“It is acceptable. As I was struggling, it was like a protection. So, I would not fall down.”* [IDI woman, 23 years old, rural]

However, some women answered that they could not accept being physically restrained because they wanted to feel free during labor, which may already be painful.

*“I wouldn’t like it as I was in labor. I wanted to feel free. If they tied me like that, my labor pain would be longer. I wouldn’t feel free to push out the baby.”* [IDI woman, 33 years old, rural].

Physical restraint may then limit the women’s ability or strength to push the baby out.

Regarding the providers, all of them did not accept to physically restrain the woman during labor if she was conscious and in good health. Otherwise, they agreed to physical restrain the women if needed to prevent them from falling from the delivery bed, for example, in case of pre-eclampsia/eclampsia, or when providing intravenous fluids.

*“No, I would not. There is no reason to accept it. If a patient delivers in a normal way, we do not hold by hands or we do not accept it. At that time, there is one thing, we ask her to hold the delivery bed and to push her legs to deliver. We do not have to control them. But the patients have fit or severe [pre-eclampsia], the patient attendants have to hold her still. We need to control her by hand. If it is so, I would accept it.”* [IDI with a doctor]

*“At the time when she has a fit, she needs to be tied as we are worried that she will fall down. Actually, there is a position how to place a woman not to fall down. But we do not have the condition to place her like that. That is why she has to be tied. If she is given drip, we need to control her. Every moment can cause a change. As she is being given drip, in normal time, I cannot accept it. If I experienced it, I would not accept it.”* [IDI with a doctor]

Physical restraint was therefore acceptable only in situations where the providers felt the woman was at risk of hurting herself by falling off the bed during a seizure or to keep her still while receiving intravenous fluids or medication.

### Gender dynamics and power relations contributing to women’s experiences of mistreatment during childbirth

The findings in the following sections are presented using a gender framework analysis approach [27] to explore how gender dynamics and power relations contribute to women’s experiences of mistreatment during childbirth. Gender dynamics are presented according to access to resources, division of labor, social norms, and rules and decision-making to understand gender as a driver of inequality and power relations. How power is shaped and negotiated by individuals, structures and the environment are explored throughout each of these sections. Table 5 presents the gender analysis framework, and Additional file 1 presents the gender analysis framework in more detail.

**Table 5:** Gender analysis framework to understand how gender dynamics and power relations contribute to women’s experiences of mistreatment during childbirth (Additional file 1 for extended version). In Myanmar facilities, women are first admitted to the labor ward when they come for childbirth but not ready for birth. Women are moved to the waiting room when they are experiencing contractions and in the early stage of labor, and to a separate delivery room when they are in advanced labor. After birth, women are either moved to a separate postnatal room, or back to the labor ward (if no separate postnatal room)

**Access to resources**	Financial resources	– Facility-based childbirth care free-of-charge – Informal costs (gate attendants, lower-level health workers, food/gifts to providers to express gratitude for care – Indirect costs medicines, consumables and equipment
	Access to information	– Women with lower health literacy may not understand explanations/instructions from healthcare providers, leading to misunderstanding. – When women arrive for childbirth with no antenatal care, without early/regular antenatal care, or without medical records, there may be conflict with providers
	Support from family companions	– Intermittent support from female family companions only in labor ward and only for essential tasks in waiting room. – Male companions allowed only in labor ward and only during visitor hours (up to 4 h per day) – No family companions allowed in delivery room
	Human resources	– High patient-to-provider ratios limit interaction time between women and providers – Insufficient salaries for providers for long hours, overtime, and additional tasks (clerical work, management) – Insufficient number of lower-level hospital staff (cleaner, gate keeper), and not trained as a professional cadre
	Health facility structure & conditions	– Multiple beds in the same room (labor ward, waiting room, delivery room, and postnatal rooms) with no curtains or partitions – Insufficient number of beds in the labor ward, and the waiting room to accommodate the patient load – Separate rooms for waiting room, delivery room, and postnatal ward; women move between rooms throughout labor and birth – Need for clean and reliable bathrooms, water supply, and electricity
**Division of labor**	Woman-level	– Women expected to attend antenatal care, arrive at the facility “on time” for the birth, for their personal hygiene (before examination, and understand and “obey” the rules of the health facility and the instructions from the providers
	Family-level	– Family companions expected to care for women (food, drinks, change of clothes, prayer, and encouragement), clean up after the birth, listen to explanations from providers, communicate this information to other family members, and obey the rules of the health facility
	Provider-level	– Community-based midwives and public health staff expected to educate women before arrival at health facility – Providers expected to provide emotional support and clinical care, and effectively communicate with women and their families about care provided, how family companions can support, and any additional fees – Nurses expected to supervise cleaners and lower-level providers, control the flow of family companions to prevent crowdedness – Providers expected to work in unity according to the roles of their cadre; human resource constraints can make this challenging – Supervisors responsible for decision-making about care (as needed), continuous supply of medicine and equipment, managing workloads, and supervision of staff and trainees – Administrators expected to resolve inconveniences hindering work of providers, and smooth functioning of facility
	System-level	– The Ministry of Health and Sports expected to provide a reliable supply of medicines, equipment, and health workforce
**Social norms**	Choice of birthplace	– Most women prefer home birth because of the convenience, lower cost, and easier arrangements – Some women prefer hospital birth because it is perceived as safe, responsive to complications, and positive view of doctors and medicine – Women may choose hospital birth if they have higher risk health conditions, or for their first birth but not subsequent births
	Mode of birth	– Some women prefer vaginal birth as it is considered normal and safe. – Some women prefer caesarean birth to avoid labor pain
	Companionship	– Many women preferred female family companions, and preferred companionship continuously throughout labor and childbirth.
	Acceptability of mistreatment	– Most women view mistreatment was unacceptable, as it made them feel sad, worried and scared. Some women believed that it is acceptable as a method of encouragement or protection, or if it was done for the woman’s sake. – Most providers believe that mistreatment was unacceptable, but some felt that it was acceptable if used for the sake of the women. – Women and providers suggest that mistreatment may happen when women do not follow the hospital rules or provider instructions.
	Relationship with staff	– Relationships between women and the lower level staff were negative, but more positive with the doctors and nurses – Women and family companions may not understand the challenges faced by healthcare providers
**Rules and decision-making**	Factors influencing decision-making	– Most women reported that they decided where they would give birth; some influenced by family, providers during antenatal care, or by their health condition, financial situation or the distance to the hospital.
	Compliance & obedience	– Women attending antenatal care early/regularly treated with more respect as they are perceived to be prepared for the birth – Women who “comply” with the rules of the hospital and with the providers’ instructions may have better experiences – Nurses/low-level hospital staff responsible for enforcing rules (e.g. asking family companions to leave labor ward) and may become frustrated with repeatedly enforcing the same rules
	Organization of services and care	– Formal cost of facility-based childbirth is free, but informal payments are sometimes needed; women who make these informal payments may have better experiences – Males allowed on the labor ward only during visitor hours; females allowed on labor ward and waiting room intermittently; no one allowed in delivery room – Women allowed to give birth in lithotomy position, as this is how providers are trained and delivery beds are designed – Women allowed to mobilize while in the labor ward and waiting room but not delivery room – Women allowed to eat and drink in the labor ward and waiting room but not delivery room.

### Access to resources

Access to resources refers to the financial resources, access to information, support during labor and childbirth, human resources, and health facility structure and conditions. Access to financial resources includes direct costs for women for transportation from home to hospital, indirect costs (to purchase medicine, consumables and equipment that are not free-of-charge), and informal costs (access to the facility and labor ward, food or gifts to providers to express gratitude for care. At times, tension may arise related to these costs, such as if a woman believes childbirth to be free-of-charge but is then requested to purchase medication or provide gifts to receive better care. Limited access to information and low health literacy by women can lead to misunderstanding and conflict between the women and the providers, as these women may struggle to understand explanations or instructions from healthcare providers. Women who did not attend any antenatal care, did not receive early or regular antenatal care, or did not bring medical records with them to the hospital may be treated differently by providers, who perceive them as not being prepared.

*“When I told them I am poor, I was asked to buy medicine for 10000ks for that day. … . We also had spare meal cost. … .finally we had to borrow money from the other with daily interest. I haven’t solved the debt till now.”* [FGD, woman, urban]

*“The Government has been providing medicines. But, when we are actually in need, we are run out of medicine. … Attendants complain that medicine is being provided and why they need to buy it.”* [IDI with a nurse]

There were some constraints for women to receive continuous support during labor and childbirth from family companions. In studied facilities in Myanmar, women are first admitted to the labor ward when they arrive for childbirth. Women are moved to the waiting room when they are experiencing contractions and in the early stage of labor, and to a separate delivery room when they are in advanced labor. After birth, women are either moved to a separate postnatal room, or back to the labor ward (if no separate postnatal room exists or is unavailable). Only female family members were allowed during certain times in early labor (labor ward) and to provide food and drinks in the waiting room. Male companions could only be present during visitor hours (up to 4 h per day) in the labor ward only. No family companions were allowed in the delivery room.

*“Only one attendant was allowed to stay. They had to go out when the doctors had round check … At night, my mom attended me. My husband could not attend me. Male could not attend in the woman ward.”* [IDI woman, 25 years old, urban]

*“If we allow an outsider (a patient attendant) to come in, we have to worry about infection. In the present condition, we allow patient attendants to come into the waiting room, not into the delivery room.”* [IDI with a doctor]

Human resource constraints included high patient-to-provider ratios, insufficient salaries, no specific training for lower-level hospital staff, and insufficient cleaners and other non-provider staff. Health facility structures and conditions contributing to how women are treated include multiple beds in the same room, insufficient number of beds to accommodate patient load, and no curtains or partitions to provide privacy. Clean and reliable bathrooms, water supply, and electricity were also needed. There are separate rooms for early labor (waiting room), advanced labor to childbirth (delivery room) and after childbirth (postnatal ward) and women are required to move between these rooms, which may be challenging and uncomfortable when they are experiencing labor pains or immediately postpartum.

*“Much work load. At first, there were only three doctors here. It was not balance. We had to take night duty successively. So, when it happened many days, we became tired and were not in good mood.”* [IDI with a doctor]

### Division of labor

Division of labor is across multiple levels: women, family, provider and system. Providers expected women to attend antenatal care to prepare for birth, to arrive at the hospital “on time” for birth and to be responsible for their own personal hygiene before examinations. Family members were expected to care for the woman, and to clean up after childbirth (windows and floors as necessary). Both women and their family members were expected to “obey” the rules of the facility and the instructions from the providers. Providers are responsible to provide clinical care and health education for women using effective communication. Different levels of hospital staff and cadres were responsible for fulfilling their different roles in unity to ensure smooth functioning of the hospital. The Ministry of Health and Sports is expected to provide reliable supply of medicines, equipment, and health workforce. When these roles are not fulfilled or inadequately fulfilled, conflict may arise that may impact how women are treated.

*“Since women have pregnancy, I want them to have their decision, where to deliver, how to deliver and so on. I want them to think of it in advance. .. Since they have the first antenatal visit, I think they should have thought how to take antenatal care, to whom they should go, with whose help they would deliver, where they would deliver, how they would deliver, if they would deliver via vagina or with CS.”* [IDI with a doctor]

*“As they instructed, we had to stay clean. We had to do as they said. If we buy what they asked to, if we obeyed what they said, we would be treated well.”* [IDI woman, 27 years old, rural]

### Social norms

Social norms included preferences for place and mode of birth, preferences for labor companionship, acceptability of mistreatment and relationships between women and staff. Most women preferred home birth because of the perceived convenience, lower cost, and easier support by family members. Some women preferred hospital birth either for their first birth (but not subsequent births which they viewed as lower risk), or if they had a higher risk condition as they perceived that it was safer and better able to respond to potential complications. Most women preferred vaginal birth as it was considered normal and safe, while a minority of women preferred caesarean birth to avoid labor pain. Many women preferred to have female family members present continuously throughout labor and childbirth to care for and support them.

*“If I delivered normally, I didn’t have the plan to deliver at the hospital. My mom also said to deliver at home as they were around me. My friends also delivered at home. As for me, it was the first baby and it was up right position, I was afraid and delivered at the hospital.”* [IDI woman, 29 years old, urban]

Most women believed that mistreatment was not acceptable as it made them feel sad, worried and scared. However, some women perceived that pinching, slapping, shouting, and physical restraint was acceptable if it was used to encourage or protect women. Although most providers believed that pinching, slapping, shouting, and physical restraint were unacceptable, some felt that they had to do use these measures for good purposes or for the sake of the women. Women and providers suggest that mistreatment may happen when women do not follow the rules of the hospital or instructions from the providers. Women perceived that their relationship with providers (doctors and nurses) was positive, but relationship with other staff (ward guard and cleaners) was problematic. Some providers believed that women and their families did not understand the challenges faced by providers. These social norms frame the context and conditions of how women give birth in Myanmar and influence individual interactions between the woman, provider, and birth environment.

*"The low level staffs were very bad, the doctors and the nurses were good. … Those low level staff were shouting and yelling. Everybody could understand if they said properly. So, people didn’t want to talk to the guards. And that guard man, if we gave him ks500, he opened the gate."* [FGD, women, rural]

### Rules and decision-making

Most women decided their place of childbirth by themselves, but some were influenced by their family members (husband/partner, parents, aunt), or providers during antenatal care. Their health condition, financial situation and distance to the hospital also influenced their choice of birth place.

*“If it was easy, I planned to deliver at home. Delivering at home costs less. If I had 30000ks or 40000ks, it would be alright. I had planned to deliver at home.”* [FGD, woman, rural]

Women may have better experiences if they “comply” with the rules of the hospital, with the providers’ instructions, and attend early and regular antenatal care. Hospital staff responsible for enforcing rules may become frustrated when family members repeatedly do not follow the hospital rules.

Formal cost of facility-based childbirth is free, but informal payments may be necessary; women who make these informal payments may have better experiences.

*“I had to pay for the cleaning fee. It was as much as I wished. For the private room I had to pay 9000ks. Cups for keeping medicine were sold to us. Only 500-1000ks for trolley. I had to have oxygen tank. I had to borrow it outside. I had to pay for the blood test.”* [IDI woman, 36 years old, urban]

Informal rules allow only intermittent support from family companions, including from males only in the labor ward during visiting hours, and from females intermittently on the labor ward and waiting room. Women were allowed to mobilize, eat and drink while in the labor ward and waiting room, but not in the delivery room and family members provided any necessary food and drink. These formal and informal rules govern the context in which women give birth and how decisions are made during labor and childbirth.

## Discussion

This is the first qualitative study in Myanmar to explore mistreatment of women during childbirth. While all four of the scenarios of mistreatment (pinching/slapping, shouting at, physical restraint and refusing to help) were found to be unacceptable for most women, providers, and administrators, there are some reasons and conditions in which some scenarios of mistreatment were considered reasonable responses. Physical restraint during labor was acceptable for women if it was perceived to be intended to speed up labor or to provide protection or encouragement for the woman to have a good outcome. Providers and administrators believed that physical restraint may be necessary to prevent women from falling off the bed in case of pre-eclampsia. Pinching or slapping was acceptable for women and providers if it was used for encouragement or for their sake. These findings on conditions where mistreatment may be acceptable are in accordance similar findings in West Africa (Guinea and Ghana) where these type of mistreatment behaviors were not acceptable for most women unless used to save the life of the woman [17, 21]. Furthermore, in Nigeria and Ghana [19, 21] some women and providers considered these mistreatment behaviors as appropriate and acceptable measures to gain compliance from the woman, correct perceived “disobediences”, and ensure a good outcome for the baby. In our study, some scenarios were found to be more unacceptable for women when their emotions would be affected (feeling sad or angry); for example being shouted at or being refused help during labor.

To our knowledge, this is the first use of gender analysis to explore how gender dynamics and power relations contribute to women’s experiences of mistreatment during childbirth. Firstly, both providers and women expected women to understand and “obey” the rules of the health facility and instructions from providers in order to have better experiences. In this way, deviation from following the rules and instructions puts the providers in a place where perpetrating acts of mistreatment were justifiable. Providers expressed that when women are “disobedient” or “uncooperative,” then they are to be blamed for being mistreated. Women with lower health literacy may be in a more vulnerable position as it may be more difficult for them to understand explanations or instructions from providers. These findings are aligned with Morgan et al’s gender analysis of maternal health in Uganda, which found that poor behaviors by providers could be disempowering for women, particularly during the vulnerable time of childbirth, and marginalized women may be more likely to experience poor treatment [29].

In Myanmar, there are only 14 healthcare providers per 10,000 people with necessary midwifery skills, despite WHO recommending at least 23 per 10,000 to achieve 80% coverage of skilled birth attendance [30, 31]. High patient-to-provider ratios, and insufficient salaries for providers for long hours, overtime, and additional task were reported in our study, and these conditions may exacerbate frustration and stress of providers and constrain the provision of quality care. These findings are similar to a study in Guinea where it was reported that mistreatment occurred because providers were poorly trained and overworked [18].

In our study, we found that the physical structure and organization of the healthcare facility may not create favorable conditions to provide a quality care for the women. For example, privacy is challenging to maintain when there are multiple beds in the same room (labor ward, waiting room, delivery room, and postnatal rooms) with no curtains or partitions. Furthermore, women are required to move themselves and any belongings between these four rooms as their labor progresses, which may be challenging or uncomfortable, particularly in the later stages of labor. Different rules govern a woman’s access to companionship throughout each room including the gender of the companion and the time they are allowed to be present (continuously vs intermittently). Critical reflection on the physical design and organization of maternity services, for example by using human-centered design approaches, may be a valuable approach to ensure that services are useful, desirable, and usable to the woman and her family, while ensuring efficiency and effectiveness to the provider [32] . Using human-centered design approaches may also be a useful tool to implement effective interventions, including continuous support for women during labor and childbirth [33], particularly where there may be resistance from providers [34]. Continuous support has been demonstrated to improve women’s experiences of childbirth [33], particularly related to improving communication, emotional support and respect and dignity. The Myanmar Ministry of Health and Sport should consider the importance of companionship throughout labor and childbirth and how to implement effectively and sustainably across facilities in Myanmar.

Establishing quality improvement processes for health care services is challenging for resource-limited settings, and both clients’ and healthcare providers’ participation is crucial for implementation. The findings of this study reflect the current condition related to social norms, power dynamics and acceptability of mistreatment, and can be used as a starting point to consider how women and providers perceive mistreatment behaviors differently. Given that women’s perceptions of quality of care influence their future healthcare choices [4] and that mistreatment during childbirth can amount to human rights violations [35], it is critical to consider how best to address the situation of mistreatment during childbirth in Myanmar. Women should be empowered more to be able to understand their right for dignified and respectful childbirth, and their right to receive quality health care. It is important for the women to know when they were mistreated and aware that any kind of mistreatment is unacceptable. Moreover, an effective accountability mechanism should be developed for the women to give feedback for the care they received, and this may contribute to reduce mistreatment.

### Strengths and limitations of the study

The strengths of the study were being the first study on the mistreatment of women during childbirth in Myanmar. Including the perspectives of both women and providers is important to have a more holistic perspective of the situation and power dynamics affecting maternity care. Using the gender analysis framework approach allowed us to consider gender dynamics and power relations contribute to women’s experiences of mistreatment during childbirth. This study also has some limitations. This study was conducted in two hospitals of Bago Division, and it is possible that women and healthcare providers from other areas of the country may have different experiences and different views on these issues; therefore, potentially limiting the transferability of our research findings. There is limited research evidence available on maternal health in Myanmar, particularly related to quality of care, which limited our ability to compare and contrast our study findings to other areas. Furthermore, mistreatment is a challenging topic to discuss with providers in particular (social desirability bias); we note that despite this challenge, providers did discuss their own experiences and factors influencing how women are treated during childbirth.

## Conclusion

Eliminating all forms of mistreatment of women during childbirth is essential to increase delivery by skilled birth attendants and facility-based childbirth by ensuring quality maternity care services. These findings have contributed to the development of tools to measure mistreatment [22]. Understanding both women’s and providers’ perceptions and acceptability of various forms of mistreatment is an important initial step and findings from this study will be disseminated in effective ways both nationally and internationally. The results will be shared with various local stakeholders, including Ministry of Health and Sports, hospitals, communities and various local and international non-governmental organizations to raise awareness and to catalyze action to reduce mistreatment during childbirth.

## Supplementary information

**Additional file 1.** Gender framework analysis table (Extended Version).

## Data Availability

Full qualitative data are available upon reasonable request from the corresponding author.

## Abbreviations

DMR: Department of Medical Research
FGDs: Focus Group Discussions
IDIs: In-depth Interviews
LMICs: Low and middle-income countries
MDHS: Myanmar Demographic and Health Survey
MMR: Maternal mortality ratio
TFR: Total Fertility Rate

## References

[1] WHO; UNICEF; UNFPA; World Bank Group and the United Nations Population Division (2015). Trends in maternal mortality: 1990 to 2015.

[2] Alkema L, Chou D, Hogan D, Zhang S, Moller A-B, Gemmill A (2016). National, regional, and global levels and trends in maternal mortality between 1990 and 2015 with scenario-based projections to 2030: a systematic analysis by the United Nations Maternal Mortality Estimation Inter-Agency Group. Lancet (London, England).

[3] WHO, UNICEF, UNFPA, World Bank Group and the United Nations Population Division. Trends in maternal mortality 2000 to 2017: Geneva: World Health Organization; 2019.

[4] Bohren MA, Hunter EC, Munthe-Kaas HM, Souza JP, Vogel JP, Gülmezoglu AM (2014). Facilitators and barriers to facility-based delivery in low- and middle-income countries: a qualitative evidence synthesis. Reprod Health.

[5] Babalola S, Fatusi A (2009). Determinants of use of maternal health services in Nigeria - looking beyond individual and household factors. BMC Pregnancy Childbirth.

[6] WHO (2015). The prevention and elimination of disrespect and abuse during facility-based childbirth. WHO statement: Every woman has the right to the highest attainable standard of health, which includes the right to dignified, respectful health care.

[7] Bhutta ZA, Salam RA, Lassi ZS, Austin A, Langer A. Approaches to improve the quality of maternal and newborn health care: conclusions, evidence gaps and research priorities. Reprod Health. 2014;11(Suppl 2):1–9. 10.1186/1742-4755-11-S2-S5 PMC4160923 25208572

[8] United Nations Population Fund (UNFPA). International Conference on Population and Development. Available at https://www.unfpa.org/icpd. Accessed 11 July 2019.

[9] Tunçalp Ӧ, Were WM, MacLennan C, Oladapo OT, Gülmezoglu AM, Bahl R (2015). Quality of care for pregnant women and newborns-the WHO vision. BJOG..

[10] Redshaw M, Hockley C (2010). Institutional processes and individual responses: Women’s experiences of Care in Relation to cesarean birth. Birth..

[11] United Nations General Assembly (1976). The international covenant on economic, social and cultural rights.

[12] Kruger L, Schoombee C (2010). The other side of caring: abuse in a south African maternity ward. J Reprod Infant Psychol.

[13] El-Nemer A, Downe S, Small N (2006). ‘She would help me from the heart’: an ethnography of Egyptian women in labour. Soc Sci Med.

[14] WHO. Standards for improving quality of maternal and newborn care in health facilities. Geneva: World Health Organization; 2016.

[15] Bohren MA, Vogel JP, Hunter EC, Lutsiv O, Makh SK, Souza JP (2015). The mistreatment of women during childbirth in health facilities globally: a mixed-methods systematic review. PLoS Med.

[16] Vogel JP, Bohren MA, Tunçalp Ö, Oladapo OT, Adanu RM, Baldé MD (2015). How women are treated during facility-based childbirth: development and validation of measurement tools in four countries – phase 1 formative research study protocol on behalf of the WHO research group on the treatment of women during childbirth. Reprod Health.

[17] Balde MD, Bangoura A, Diallo BA, Sall O, Balde H, Niakate AS (2017). A qualitative study of women’s and health providers’ attitudes and acceptability of mistreatment during childbirth in health facilities in Guinea. Reprod Health.

[18] Balde MD, Diallo BA, Bangoura A, Sall O, Soumah AM, Vogel JP (2017). Perceptions and experiences of the mistreatment of women during childbirth in health facilities in Guinea: a qualitative study with women and service providers. Reprod Health.

[19] Bohren MA, Vogel JP, Tunçalp Ö, Fawole B, Titiloye MA, Olutayo AO (2016). “By slapping their laps, the patient will know that you truly care for her”: a qualitative study on social norms and acceptability of the mistreatment of women during childbirth in Abuja, Nigeria. SSM Popul Heal.

[20] Bohren MA, Vogel JP, Tunçalp Ö, Fawole B, Titiloye MA, Olutayo AO (2017). Mistreatment of women during childbirth in Abuja, Nigeria: a qualitative study on perceptions and experiences of women and healthcare providers. Reprod Health.

[21] Maya ET, Adu-Bonsaffoh K, Dako-Gyeke P, Badzi C, Vogel JP, Bohren MA (2018). Women’s perspectives of mistreatment during childbirth at health facilities in Ghana: findings from a qualitative study. Reprod Health Matters.

[22] Bohren MA, Vogel JP, Fawole B, Maya ET, Maung TM, Diouldé Baldé M (2018). Methodological development of tools to measure how women are treated during facility-based childbirth in four countries: labor observation and community survey. BMC Med Res Methodol.

[23] Bohren MA, Mehrtash H, Fawole B, Maung TM, Balde MD, Maya E, et al. How women are treated during facility-based childbirth in four countries: a cross-sectional study with labour observations and community-based surveys. Lancet. 2019;394(10210):1750–63. 10.1016/S0140-6736(19)31992-0 PMC6853169 31604660

[24] UNFPA and Ministry of Health and Sports. Myanmar Midwifery Situation 2014 Synthesis Report. Nay Pyi Taw, The Republic of Union of Myanmar: Ministry of Health and Sports; 2014.

[25] Ministry of Health and Sports (2017). Myanmar Demographic and Health Survey 2015–16.

[26] Braun V, Clarke V (2006). Using thematic analysis in psychology. Qual Res Psychol.

[27] Morgan R, George A, Ssali S, Hawkins K, Molyneux S, Theobald S (2016). How to do (or not to do) … gender analysis in health systems research. Health Policy Plan.

[28] Ritchie JSL, BR BA (1993). Qualitative data analysis for applied policy research. Analysing Qualitative Data.

[29] Morgan R, Tetui M, Muhumuza Kananura R, Ekirapa-Kiracho E, George AS (2017). Gender dynamics affecting maternal health and health care access and use in Uganda. Health Policy Plan.

[30] World Health Orgainzation. The World Health Report 2006 - working together for health. Geneva: World Health Organization; 2013.

[31] Ministry of Health Myanmar (2012). Health Workforce Strategic Plan 2012-2017.

[32] Salgado M, Wendland M, Rodriguez D, Bohren MA, Oladapo OT, Ojelade OA (2017). Using a service design model to develop the “passport to safer birth” in Nigeria and Uganda. Int J Gynecol Obstet.

[33] Bohren MA, Hofmeyr GJ, Sakala C, Fukuzawa RK, Cuthbert A (2017). Continuous support for women during childbirth. Cochrane Database Syst Rev.

[34] Bohren MA, Berger BO, Munthe-Kaas H, Tunçalp Ö. Perceptions and experiences of labour companionship: a qualitative evidence synthesis. Cochrane Database Syst Rev. 2019. 10.1002/14651858.CD012449.pub2 PMC6422112 30883666

[35] Khosla R, Zampas C, Vogel JP, Bohren MA, Roseman M, Erdman JN (2016). International human rights and the mistreatment of women during childbirth. Health Hum Rights.

[36] Tong A, Sainsbury P, Craig J (2007). Consolidated criteria for reporting qualitative research ( COREQ ): a 32-item checklist for interviews and focus groups. Intern J Qual Heal Care.

