# Supplementary material for: A qualitative study on acceptability of the mistreatment of women during childbirth in Myanmar
Source: Reprod Health. 2020 Apr 20;17:56. doi: 10.1186/s12978-020-0907-2 (PMC7171855; doi:10.1186/s12978-020-0907-2)
Supplement: Supplementary file 1 — Additional file 1. Gender framework analysis table (Extended Version). [file 12978_2020_907_MOESM1_ESM.docx]

Additional file 1. Gender framework analysis table to understand how gender dynamics and power relations contribute to women’s experiences of mistreatment during childbirth. This table presents the full results of the gender framework analysis. In facilities in Myanmar, women are first admitted to the labor ward when they arrive at the facility for childbirth, where their labor progression is typically established with a vaginal examination and medical history. Women are moved to the waiting room when they are experiencing contractions, and onto a separate delivery room when they are in advanced labor. After the birth, women are either moved to a separate postnatal room, or back to the labor room if there is no separate postnatal room.

| Gender dynamics and power relations | | | Perspective | |
| --- | --- | --- | --- | --- |
| Access to resources | | | | |
| Financial resources | | Women and families were responsible for costs associated with facility-based childbirth, including for transportation from home to facility, informal costs (to gate attendants to access the facility, to lower-level health workers to access the labor ward and to be treated better), to give food or gifts to providers to express gratitude for care, and to purchase medicines, medical consumables and equipment (not provided free-of-charge from the hospital). | | Women and providers |
| Access to information | | Some women (e.g. less educated, or from ethnic/linguistic minorities or remote communities) may have lower health literacy and struggle to understand explanations or instructions from healthcare providers, which can lead to misunderstandings. | | Providers |
|  |  | Some women arrive to the facility for childbirth without attending any antenatal care, or without early/regular antenatal care, or without medical records (e.g. of their expected due date or ultrasound results), which can lead to conflict with providers. | | Providers |
| Support during labor & childbirth from family companions | | In the labor ward, women may receive intermittent support from female family companions, and typically only one companion is allowed per woman. Female companions are required to leave during ward rounds and at any other time requested by providers. In the waiting room, female family attendants are allowed as necessary, typically for essential tasks such as providing food or drinks to the woman. In the delivery room, no female family companions are allowed. | | Women and providers |
|  |  | Males, such as husbands or male partners, are only allowed on the labor ward during visitor hours (typically up to four hours per day during two periods). Males are not allowed in the waiting room or delivery room. | | Women and providers |
| Human resources | | High patient-to-provider ratios limit the interaction time between women and providers | | Women and providers |
|  |  | There are insufficient salaries for providers to compensate for long hours, overtime, and additional tasks (such as form filling, management | | Providers |
|  |  | The lower-level hospital staff (e.g.: ward guards, cleaners )are recruited as daily, casual workers, then they became permanent workers,. However, they are not trained as a professional cadre and there is no specific training for them. | | Providers |
|  |  | There is an insufficient number of cleaners and other non-provider hospital staff to run the hospital efficiently. | | Women and providers |
| Health facility structure & conditions | | There are multiple beds in the same room in the labor ward, waiting room, delivery room, and postnatal rooms. | | Women and providers |
|  |  | There is an insufficient number of beds in the labor ward, and the waiting room to accommodate the patient load | | Women and providers |
|  |  | There are separate rooms for the waiting room (early labor), delivery room (advanced labor to childbirth) and postnatal ward (after childbirth), and women have to move between the rooms. | | Women and providers |
|  |  | There are no curtains or partitions to provide privacy during examinations, cleaning, or childbirth (waiting room, labor wards and delivery rooms), except if women pay for a private room. Some providers mentioned that in some facilities, there are examination rooms that provide privacy during examination. | | Women and providers |
|  |  | There is a need for clean and reliable bathrooms, water supply, and electricity supply. | | Women |
| Division of labor | | | | |
| Woman-level | | Women are expected to attend antenatal care to prepare for the birth and to arrive at the facility “on time” for the birth. | | Providers |
|  |  | Women expected to understand and “obey” the rules of the health facility and the instructions from the providers. | | Women and providers |
|  |  | Women are responsible for their own personal hygiene (e.g. before examination). | | Providers |
| Family-level | | Family companions are expected to care for women during their stay in the health facility by providing food, drinks, change of clothes, prayer, and encouragement | | Women and providers |
|  |  | Family companions are expected to clean up after childbirth (windows and floor as necessary).Women without companions are expected to clean up by themselves after birth. | | Providers |
|  |  | Family attendants are expected to listen to explanations from providers, communicate this information to other family members, and obey the rules of the health facility | | Providers |
| Provider-level | | Providers are expected to provide both emotional support and clinical care, encouragement, teach women how to push, and support with breastfeeding. | | Women and providers |
|  |  | Providers are expected to effectively communicate to women and their families about care provided, how family companions can support, and any additional fees needed (e.g. for medication), or use ward funds to purchase medication for poor women | | Providers |
|  |  | Nurses are expected to supervise cleaners and lower-level providers. | | Providers |
|  |  | Nurses and other hospital staff are expected to control the flow of family companions so that the ward is not overloaded | | Providers |
|  |  | Providers are expected to work in unity with each other according to their roles at each level, however human resource constraints can make it challenging to achieve unity. | | Providers |
|  |  | Supervisors are responsible for providing continuous supply of medicines and equipment, managing workload of other providers, and supervisin staff and trainees | | Providers |
|  |  | Supervisors are expected to make decisions about care when needed | | Providers |
|  |  | Hospital administrators are expected to solve any inconveniences that can hinder the work of the providers, and are responsible for the facility to be functioning smoothly | | Providers |
|  |  | Community-based midwives and public health staff are expected to educate women before they arrive to the health facility (e.g. during antenatal care, health education, and referral) | | Providers |
| System-level | | The Ministry of Health is expected to provide a reliable supply of medicines, equipment, and health workforce | | Providers |
| Social norms | | | | |
| Choice of birthplace | | Most women prefer home birth because of the convenience, lower cost, and easier arrangements (e.g. do not need to organize for family members to attend at the facility) | | Women and providers |
|  |  | Some women prefer giving birth in the hospital because it is perceived as safe, able to respond to complications, and the presence of doctors and medicines is viewed positively. Some women believe that childbirth at home is risky. | | Women and providers |
|  |  | Women may choose to give birth in the hospital if they have a higher risk condition (e.g. pre-eclampsia, placenta previa, breech presentation, older age) or for their first birth but not subsequent births. | | Women and providers |
| Preference for mode of birth | | Some women prefer vaginal birth as it is considered normal and safe, and most women had vaginal birth. | | Women and providers |
|  |  | Some women prefer caesarean birth particularly to avoid labor pain, but they are encouraged by providers to try vaginal birth | | Women and providers |
| Preferences for family companions | | Many women preferred female family companions to care for them and if allowed, they preferred companionship continuously throughout labor and childbirth, rather than intermittently in the waiting room and delivery room. | | Women |
| Acceptability of mistreatment | | Most women view mistreatment as unacceptable behavior, as it made them feel sad, worried and scared. Some women believed that pinching, slapping, shouting at, or physically restraining a woman was acceptable as a method of encouragement or protection, or if it was done for the woman’s sake. | | Women |
|  |  | Most healthcare providers believed that pinching, slapping, shouting at, or physically restraining a woman were unacceptable. However, some expressed that they felt that they had to do use these measures, but were used for good purposes for the sake of the women. | | Women and providers |
|  |  | Women and providers suggest that mistreatment may happen when women do not follow the rules of the hospital or instructions from the healthcare providers | | Providers |
| Relationship with staff | | Relationships between women and the lower level staff were negative, but more positive with the doctors | | Women |
|  |  | Women and family companions may not understand the challenges faced by healthcare providers. | | Providers |
| Rules and decision-making | | | | |
| Factors influencing decision-making | Most women reported that they decided where they would give birth, and some were influenced by family members (husband/partner, parents, aunt), healthcare providers during antenatal care, or by their health condition, financial situation or the distance to the hospital | | | Women and providers |
| Compliance & obedience | Women who attend antenatal care early and regularly will be treated with more respect as they are perceived by providers to be prepared for the birth. | | | Providers |
|  | Women who “comply” with the rules of the hospital and with the providers’ instructions may have better experiences. | | | Women and providers |
|  | Nurses and low-level hospital staff are responsible for enforcing rules (e.g. asking family companions to leave the labor ward), and may become frustrated with repeatedly enforcing the same rules. | | | Providers |
| Organization of services and care | Formal cost of facility-based childbirth is free, but informal payments are sometimes needed (e.g. tips to guards, cleaners, and lower-level providers, or cost of medication during stock-out). Women who make these informal payments may have better experiences as a result. | | | Women |
|  | Males are allowed on the labor ward during the visitor hours only. Females allowed on labor ward and waiting room intermittently, but not delivery room. | | | Women and providers |
|  | Women are allowed to give birth in the lithotomy position (for vaginal birth), as this is how providers are trained and delivery beds are designed to support the lithotomy position. | | | Providers |
|  | Women are allowed to mobilize while in the labor ward and waiting room, but not in the delivery room. | | | Women and providers |
|  | Women are allowed to eat and drink while in the labor ward and waiting room (some mentioned not being allowed to eat solid food in the waiting room), but not in the delivery room. While in the labor ward and waiting room, the family attendants provide any necessary food and drink. | | | Women and providers |
